# Empowering adolescents with life skills education in schools – School mental health program: Does it work?

**DOI:** 10.4103/0019-5545.74310

**Published:** 2010

**Authors:** Bharath Srikala, Kumar K. V. Kishore

**Affiliations:** Department of Psychiatry, National Institute of Mental Health and Neurosciences (NIMHANS), Bangalore, Karnataka, India

**Keywords:** Adolescents, impact, life skills education, psychosocial competence, school mental health program

## Abstract

**Aim:**

Mental Health Promotion among adolescents in schools using life skills education (LSE) and teachers as life skill educators is a novel idea. Implementation and impact of the NIMHANS model of life skills education program studied.

**Materials and Methods:**

The impact of the program is evaluated at the end of 1 year in 605 adolescents from two secondary schools in comparison to 423 age, sex, socioeconomic status-matched adolescents from nearby schools not in the program.

**Results:**

The adolescents in the program had significantly better self-esteem (*P*=0.002), perceived adequate coping (*P*=0.000), better adjustment generally (*P*=0.000), specifically with teachers (*P*=0.000), in school (*P*=0.001), and prosocial behavior (*P*=0.001). There was no difference between the two groups in psychopathology (*P* - and adjustment at home and with peers (*P*=0.088 and 0.921). Randomly selected 100 life skill educator-teachers also perceived positive changes in the students in the program in class room behavior and interaction. LSE integrated into the school mental health program using available resources of schools and teachers is seen as an effective way of empowering adolescents.

## INTRODUCTION

The school mental health program (SMHP) is a very important and integral part of the educational system worldwide. In India, the SMHP is yet to be recognized and initiated as a part of the health component in schools.[1] In practice it is restricted to individual work by child mental health professionals especially in big metropolitan cities focusing on sensitization of teachers on child developmental and mental health issues. Counseling services for students with persistent emotional issues and a referral system is set up in a few urban clinics.

The educational philosophy in ancient India was one of guru-chela/shisya parampara and stressed on the teacher being responsible both for literacy/knowledge and personality development in the ward. However, education, which is currently prevalent in our country, is achievement oriented than child oriented. It does not address the needs of all the children who in spite of various levels of scholastic competence are capable of learning and need to develop those skills, and become empowered to live effectively in this world. This empowerment is very essential in today’s context in India as there is rapid globalization and urbanization with a breaking up of joint families and the traditional support systems.[2] Academic stress, violence including bullying, sexual permissiveness, easy drug availability and abuse, crowding, poor infrastructure, social divide are some of major issues which a youth has to contend with in this rapidly changing social scene of India. An empowered child has the competence to cope with the challenges of life using the available resources even amidst such adversities.

Methods to improve the psychosocial competence and resilience of the adolescent as health promotional activities and development oriented approach need to be included in the school syllabus and provided as much relevance as the Three Rs (reading, writing, arithmetic).

LSE is one such program.[3] The current study is on the impact of a LSE model as a program in secondary schools.

### Aims

To assess the impact of the life skills education program (LSE program -NIMHANS model) by assessing the difference between adolescents who were in the program and not in the program.

More specifically to assess the difference between the adolescents who were in the LSE program (NIMHANS model) for a year and the ones who were not in the program in the following areas.

1. Coping
2. Self-esteem
3. Adjustment in various areas
4. Psychopathology

### NIMHANS model

The present model of health promotion using life skills approach for adolescents in secondary schools was initiated by the authors in late 1996 but crystallized in late 2002.[4–6] It is a model which is comprehensive focusing on all developmental issues of adolescents; it uses experiential learning with peers using participatory methods thus enabling the adolescent with psychosocial skills. The model also uses the available infrastructure of the school and the teachers for implementation of the program in a continuous manner over the academic years as a co-curricular activity for maximum effect. The Model is discussed in detail elsewhere.[7]

### Life skills

Life skills (LS) are abilities for adaptive and positive behavior that enable individuals to deal effectively with the demands, challenges, and stress of everyday life. Childhood and adolescence are the developmental periods during which one acquires these skills through various methods and people.[8]

The generic LS, which need to be taught at the schools level especially to adolescents, are as follows.

- Critical thinking and creative thinking
- Decision making and problem solving
- Communication skills and interpersonal relations
- Coping with emotions and stress
- Self-awareness and empathy

### Implementation of the program as a project

Department of Public Instruction (DPI), Karnataka, in collaboration with NIMHANS planned to implement the NIMHANS model of the health promotion using LSE in four diverse districts (Bangalore rural, Bangalore urban, Udupi, Haveri) covering selected 261 secondary schools and 55,000 adolescents. Following steps were adopted in three phases for the implementation.

1. Translation of the resource materials into the local language, i.e. Kannada.[9–11]
2. Discussion with the Adolescent Education Division of the Department of State Education, Research and Training (DSERT), Karnataka, identification of master trainers in each of the identified district from District Institute of Education and Training (DIET).
3. Training of the identified 31 master trainers over 5 days in two batches on the concepts of adolescent development, challenges and opportunities in adolescence, life skills, values, LS education, facilitation, using the activities to impart LSE in classes, use of the resource materials, and training of teachers as LS educators.
4. LSE awareness workshops for the block education officers (BEOs) and the head masters of the identified schools.
5. Planning/preparatory workshops with BEOs of the identified four districts.
6. Capacity building by training of the teachers in the identified secondary schools as LS educators by the trained master trainers over 3 days. More than 1000 teachers were thus trained from 261 schools over 3 months. Evaluation of the training.
7. Implementation of the LS program in the identified secondary schools once a week for an hour over 12 to 20 sessions during the academic year.
8. Impact of the program on a sample of adolescents at the end of 1 year.

### Impact

Impact of the program at every level was assessed and evaluated. The resource materials were evaluated and field tested. Feedback for the training sessions by both master trainers and teachers were done. Impact of the training was also assessed both in the master trainers and teachers by a pre- and post-assessment evaluation.

The aim of this paper is to discuss only the impact of the program in an objective manner in a sample of adolescents who participated in the program for a year.

### Study group

The sample and control were selected from two schools in the Bangalore rural district (Chennapatna) and two schools from Udupi District. The control adolescents were selected from secondary schools in the same district as the sample group. Selection of the schools was random.

### Sample

Since all the students who were in the program for the previous 1 year in the two selected schools needed to be included, a total of 605 students were taken as the sample group.

Adolescents of both sexes 14 to 16 years studying in 8th, 9th or 10th standard in the two schools (Bangalore rural and Udupi) implementing the NIMHANS model of the LSE program the previous 1 year were selected. Informed Consent was taken from the parents as the adolescents were minors. These adolescents had undergone on an average 10 sessions of LSE classes during the previous year (minimum 5 and maximum 16 sessions)

### Control

A total of 423 students were assessed as controls. They were adolescents of both sexes, 14 to 16 years studying in 8th, 9th or 10th standard, in secondary schools not covered by the NIMHANS model of LSE (or other adolescent education program). Informed consent was taken from their parents also.

### Teachers

Out of the 1000 odd teachers who were trained as LS educators, 100 were selected randomly and their feedback on the perceived changes in their students who were in the LSE program, NIMHANS model, was compiled. The student indicators were prepared by the authors as a part of the resource material and available in the activity manuals[4–6] were used to assess the changes in the students.

### Tools used

1. *Rosenberg Scale of Self-Esteem*[12] (RSES, Rosenberg 1965). Designed to measure adolescents’ global feelings of self-worth. It has 10 first person statements and the responses are on 4 point scale of “strongly agree,” “agree,” “disagree” and “strongly disagree.” Scores range from 10 to 40, with higher scores indicating better self-esteem.
2. *Preadolescent Adjustment Scale*[13] (PAAS, Pareek *et al*. 1975). Though called preadolescent scale has been used in Indian studies with adolescents (Rao, *et al*. 1975). It assesses adjustment in five areas of home, school, teachers, peers, and general behavior. The total adjustment ranges from −46 to +34.
3. *Generalized Self-Efficacy Scale*[14] (GSES Jerusalem and Schwarzer 1995). 10 item measure perceived self-efficacy as an operative construct. Responses are made on a 4--point scale. Sum up the responses to all 10 items to yield the final composite score with a range from 10 to 40.
4. *Strengths and Difficulties Questionnaire – Self-Report Version*[15] (SDQ SRV Goodman *et al*. 1998). It is a brief behavioral screening questionnaire. Has 25 items divided over five psychological areas. The self report version is used for 11-16 adolescents.
5. *Class Room Indicators*.[4–6,9–11] A simple checklist was designed for the teachers who did the LSE classes eliciting observable changes in the class room behavior of the students before and in LSE classes.

### Procedure

Initially BEOs of the four districts were contacted and the objective assessment was discussed with them along with the DSERT AEP coordinator. Subsequently, from the list of 261 secondary schools, the two sample schools were selected. BEOs provided help to select the control schools not in the program from the same districts. Parents were contacted before-hand for consent. Assessment of the students was done by a research assistant who was not involved in the training and was blind whether a particular school was included in the program or not. After an introduction to the assessments and tools they are self-administered and completed by the students themselves. The research assistant clarified doubts pertaining to the tools if they were any.

As far as the teachers were concerned, feedback was being collected by post on a regular basis. At the end of 1 year the feedback of 100 teachers selected randomly was compiled.

### Analysis

SPSS version 10 was used for data entry and analysis. The chi-square test was used for categorical measures and Student ‘*t*’ test for continuous measures.

## RESULTS

The study groups both the sample and the control group did not differ in age and sex or socioeconomic status (*P*=0.001) as they were pre-selected from specific classes in government secondary schools. Their age ranged from 155 to 200 months (mean 175±13.8 months). 35 to 40% of the study groups were girls. There were more girls in Udupi school but was not significantly different from the other schools. 52% of the students in the sample group had participated in 10 LSE classes in the previous year; 23% in less than 10 sessions; and the remaining >10 sessions. The trained teachers were the LS educators and followed the NIMHANS model of LS program. The classes were interactive and participative.

The students in the control group had regular civic/moral/value education classes one to two sessions in a week according to government regulation. Often these classes were used for extra classes of other subjects.

### Feedback of teachers as LS educators

**Figure 1:**
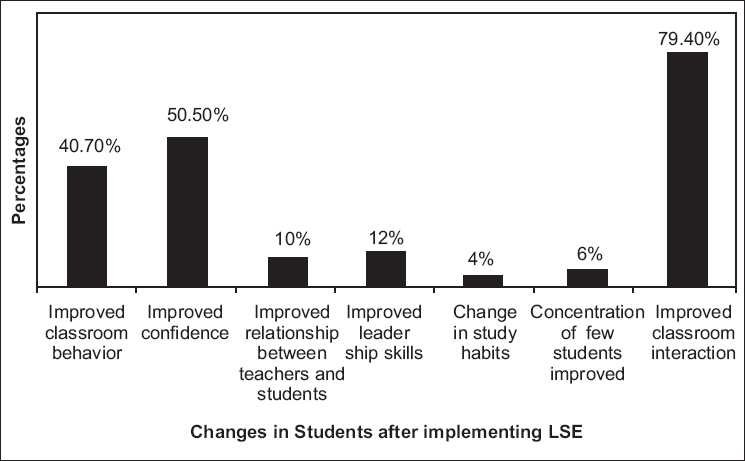
Feedback of teachers on students in the program

The 100 teachers whose feedback was compiled observed positive changes in the classroom behavior and interaction among students in their program [Figure 1].

They were not able comment on certain other indicators like decrease in the drop put rate, better performance in the academics, etc.

### Comparative assessment of students

**Figure 2:**
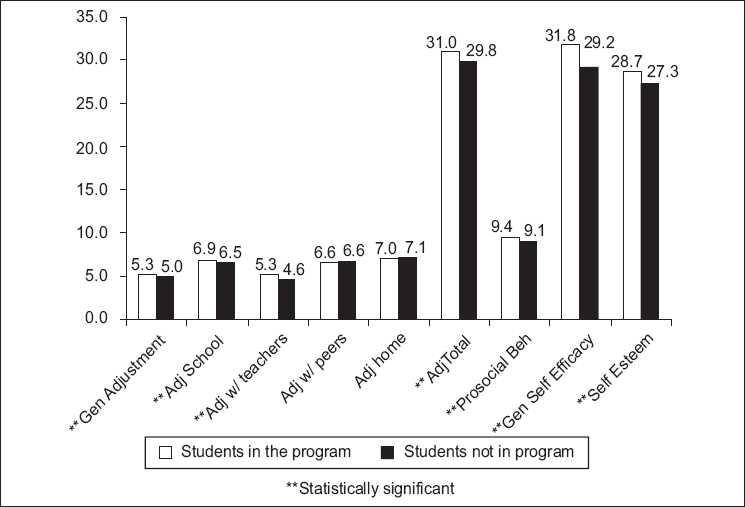
Impact of the LSE program comparision of students in the program with controls

According to their self-report, the students in the program in comparison to those not in the program were significantly better adjusted to the school and teachers; opined that they were capable of coping with issues with better self-esteem [Table 1, Figure 2].

**Table 1:** Comparison of students in the LS program with controls

Area	Sample (n-605) (mean±SD)	Control (n-423) (mean±SD)	*P* value
Adjustment (general) (PAAS)	5.26±1.10	4.95±1.30	0.000**
Adjustment in school (PAAS)	6.87±1.11	6.50±1.34	0.001**
Adjustment with teachers (PAAS)	5.30±1.15	4.63±1.29	0.000**
Adjustment with peers (PAAS)	6.63±1.23	6.64±1.43	0. 921
Adjustment in home (PAAS)	6.95±1.38	7.10±1.40	0.088
Adjustment total (PAAS)	31.02±3.92	29.83±4.79	0.000**
SDQ–emotions	4.05±2.11	4.04±2.37	0.951
SDQ–conduct	3.66±1.94	3.77±2.02	0.394
SDQ–Hyperactivity	2.44±1.35	2.57±1.41	0.143
SDQ–peers	2.68±1.48	2.64±1.58	0.670
SDQ–prosocial behavior	9.44±1.54	9.05±1.85	0.001**
SDQ–Total	12.84±4.57	13.03±5.33	0.551
General self-efficacy (GSES)	31.84±5.08	29.19±5.11	0.000**
Self-esteem (RSES)	28.66±3.6	27.28±3.6	0.002**

** Statistically Significant; PAAS - Preadolescent Adjustment Scale

SDQ - Strengths and Difficulties Questionnaire; GSES - General Self Efficacy Scale; RSES - Rosenberg Self Esteem Scale

There was no difference between the groups in adjustment with parents and peers. There was no difference between the two groups on psychopathology assessed by Strengths and Difficulties Questionnaire (SDQ).

## DISCUSSION

LSE is a novel promotional program that teaches generic LS through participatory learning methods of games, debates, role-plays, and group discussion. Conceptual understanding and practicing of the skills occur through experiential learning in a non-threatening setting. Such initiatives provide the adolescent with a wide range of alternative and creative ways of solving problems. Repeated practicing of these skills leads to a certain mastery and application of such skills to real life situation and gain control over the situation. It is a promotional program, which improves the positive mental health and self-esteem.[8] Our country places a premium on values. LS program empowers the youth to choose the appropriate values and behavior which are ingredients of positive health. LS are the processes that will make the target of values possible. The NIMHANS model of LSE was planned to be experiential, participatory and activity based for the students. “Didactic methodology” or “advice” was not part of the model at any level. Cultural sensitivity was maintained.

### Life skills education and schools

In India, education has become institutionalized. Schools need to be recognized as the single most important and recognized forum to reach out to the young population. Any program to reach the adolescents/youth has to be incorporated into the educational system to be feasible, effective, and cost-effective. In a country like ours, where resources and trained professionals are sparse and few, it is more be practical to involve and work with the teachers. The teachers are the personnel who interact with the adolescents closely. They could be trained to transfer these skills to the adolescents.[8] The methodology advised by WHO has been designed into a model by us and used in the above project. It follows a more resource-effective cascade model of training using the education set up of the country and implements the program. This methodology ensures reproducibility of the program within the existing infrastructure year after year at no extra cost. Experience also has shown that teachers need support in the form of syllabus, resource materials, and training to be able to promote LS among the adolescents. The present program has successfully incorporated the needs of the teachers and students as end users at every level/step.

Most of the programs done earlier have evaluation of implementation-money planned spent, measurement of capacity building, extent of training, and conduct of program. Impact of an effective preventive/promotional program is of paramount importance and has been discussed at length.[15] The significant strength of the present health promotion using the LS approach (NIMHANS model) has been the evaluation at every level.

Original resource materials in English and later even the translated resource materials were evaluated and modified both by expert professionals and the end users (teachers).

Trainings both at the level of the master trainers (MTs) and the teachers were evaluated both qualitatively and quantitatively-the effectiveness of training was established.

Impact of the LS program on the target population (students) is the most powerful step of the program which was planned as an integral part of the project and presented here. A total of 55,000 students in 261 secondary schools in 21 taluks of 4 districts being the target population, impact evaluation was not possible of every student before and after the LSE program. Hence impact evaluation was carried out in a sample (±500) of the target population in a comparative manner.

Since no specific assessment tools have been found to be superior to elicit LS of the adolescents, hence a set of instruments assessing assessment, presence of problems, coping and self-esteem were used. The tools were chosen such that they were self-administered, simple, earlier used in the local vernacular, and chosen diverse areas of competence-coping GSES,[14] self-esteem RSES,[12] adjustment in various areas PAAS,[13] and absence of psychopathology SDQ.[15]

Using the above tools the present comparative study indicated that even at the end of 1 year, there was a significant change in the way the adolescent perceived himself/herself in the school, with the teachers, and the confidence level of his/ability to deal with developmental challenges. One year is a very short period in development; however the model which is experiential and focused on specific issues of development seems to increase the ability of the adolescents to adjust well in the school with teachers and improve coping and self-esteem. This was also perceived by the LS educator teachers who reported better classroom behavior and interaction among the students in the program. The students in the program did not perceive better adjustment with peers than those not in the program (*P*=0. 921 and 0.670) though the LS Educator teachers reported better interaction with peers in students in the program [Figure 1]. The program probably by its facilitative and interactive nature made the adolescents more aware of their behavioral changes with the teacher and the school rather than their friends with whom they probably felt that they had always interacted well. This is evident in their reporting a difference in the prosocial behavior generally but not specifically with peers. The positive effect of LSE program in student--teacher interaction, academic performance, and peer interaction has been established by others in the West.[16–19] Parents were involved in the initial focus group discussions and later were aware of the implementation of the program. However they were not active partners in the implementation of the program. This was probably the reason for the absence of difference (*P*=0. 088) in the home adjustment between the two groups.

Perceived self-efficacy (*P*=0.000), better self-esteem (*P*=0.001), and better general adjustment (*P*=0.000) were important aspects which were significantly different between the two groups, indicating that the program prepares the adolescent to be a ‘competent’ and ‘empowered’ person in a changing, competitive, globalized world.[16]

Review indicates that most preventive program with adolescents have been specific addressing specific issues of substance abuse, teen pregnancy, violence, bullying, etc.[3,8,18] However generic programs with multiple outcomes have also been present and found to be effective too.[19] The current study confirms that absence of pre- and post-evaluation of the same students apart from a comparative group, feedback of the teachers who specifically handled the sample children, assessment of a longer nature are some of the limitations of the study.

## CONCLUSIONS

The present study of the NIMHANS model of LS program is a suitable and an effective school mental health program. The highlights of the model/program are as follows.

1. Comprehensive health including mental health through psychosocial competence in adolescents is the goal to empower the adolescent.
2. Using life skills as the medium/process.
3. Providing a structure to the program by activities.
4. Teachers as life skills educators/facilitators.

Evaluation of the Impact of the model shows that it improves adjustment of the adolescents with teachers, school, increases prosocial behavior, coping, and self–esteem, as there was a significant difference between the groups in the program and not in the program.

### Implications

Inclusion and institutionalization of SMHP using LS approach in the national mental health program (11th Five-Year Plan) and the educational policy of our country to promote psychosocial competence and reduce problem behaviors in adolescents.
